# Development of a photographic handbook to improve cystoscopy findings during resident’s training: A randomised prospective study

**DOI:** 10.1080/2090598X.2019.1596400

**Published:** 2019-04-24

**Authors:** Guglielmo Mantica, Federica Balzarini, Federico Dotta, Moises Rodriguez-Socarras, Silvia Proietti, Guido Giusti, Francesco Oneto, Marco Di Pierro, Paolo Traverso, Carlo Terrone

**Affiliations:** a Department of Urology, Policlinico San Martino Hospital, University of Genova, Genova, Italy; b Department of Urology, San Raffaele Turro Hospital, San Raffaele University, Milan, Italy; c ETCE – European Training Center in Endourology, San Raffaele Turro Hospital, Milan, Italy

**Keywords:** Cystoscopy, endourology, training, surgical training, photographic handbook

## Abstract

**Objectives**: To evaluate if the use of a photographic handbook (PH) can be a useful tool to improve the detection of disorders during cystoscopy training, as several hands-on tools have been proposed to improve technical skills but very few aim to improve specificity and sensitivity.

**Subjects and methods**: Eight junior residents (JRs) were divided into two groups: Group A, comprised four JRs with previous limited experience of performing cystoscopies; and Group B, including four inexperienced JRs who were asked to study a specific PH before performing cystoscopies. The findings of the two groups were compared using the chi-squared test.

**Results**: A total of 401 consecutive cystoscopies, of which 214 (53.4%) were performed by Group A and 187 (46.6%) by Group B, were considered. Group B showed superior ability in detecting uncommon findings (i.e., carcinoma *in situ*, bullous oedema, interstitial cystitis, etc.) with 24/46 (52.2%) detected vs eight of 32 (25%) in Group A (*P* = 0.016).

**Conclusions**: The PH was a useful tool for improving identification of pathological conditions, which could be used to enhance hands-on simulator and practical tutored training.

**Abbreviations**: CIS: carcinoma *in situ*; JR: junior resident; PH: photographic handbook; VR: virtual reality Classification: Stones/Endourology

## Introduction

Cystoscopy is one of the first operative diagnostic procedures performed by residents during their training. Several hands-on tools have been proposed to improve technical skills, but very few aim to improve specificity and sensitivity [1–3]. However, the ability to easily manipulate the cystoscope is not any more important than the ability to recognise all types of disorders during the procedure. If at first, learning how to use a cystoscope correctly is the main obstacle, later the ability to correctly identify each and every finding without the second opinion of a consultant becomes the main barrier.

We developed a photographic handbook (PH), including detailed pictures and descriptions of all usual and unusual findings during the execution of a cystoscopy, aiming to evaluate if it can be a useful tool to improve the detection of disorders during the training of a resident.

## Subjects and methods

### Study design

A randomised prospective study involving eight junior residents (JRs) was conducted from November 2017 to September 2018 in our department. The eight JRs were divided into two groups: Group A, comprised four JRs with previous limited experience performing cystoscopies (<50 cases performed); and Group B, included four inexperienced JRs who were asked to study the PH before performing cystoscopies, and after having been tested by a urology consultant. The JRs, who were blinded to the patient’s conditions, symptoms and files, had to complete a questionnaire with their findings following the execution of the cystoscopy. Afterwards, the cystoscopy, as well as the questionnaire, was also performed by a urology consultant who was not blinded to the patient’s files and symptoms. The findings of both group A and B were then compared to those identified by consultants and marked whether concordant or not. For the lesions that required it, the diagnosis was confirmed by histological examination (i.e., malignant lesion, interstitial cystitis). Patient’s randomisation was made based on the cystoscopy list scheduled for the day. Therefore, each cystoscopy was performed by a single JR under tutorship on alternate days for Group A and Group B.

All cystoscopies were performed with the same type of flexible cystovideoscope VISERA CYF TYPE V2/VA2® 16.2-F (Olympus Corp., Tokyo, Japan). As the study was conducted in a university teaching hospital, where such procedures are routinely performed by JRs under tutorship, in accordance with our Institutional Review Board an informed consent was signed by every patient before each procedure.

### Development of the PH

The PH was developed by three urology consultants (P.T, F.O, M.D.P.) in September 2017. It was divided in four different small chapters:

1. Description of the flexible cystoscope and of the instruments needed to perform a cystoscopy.
2. Description of the steps to correctly perform a cystoscopy.
3. Description of the anatomy of the bladder and urethra, including images for each normal anatomical finding.
4. Photographic description of the following disorders: BPH, bladder diverticula, urethral diverticula, bladder trabeculations, bullous oedema, double ureteric orifice, ureterocoele, aspecific inflammation, interstitial cystitis, papillary and not papillary TCC, carcinoma *in situ* (CIS), radiation cystitis, squamous cell carcinoma, schistosomiasis, urachal adenocarcinoma, foreign body, adenocarcinoma of the prostate, previous transurethral resection of bladder tumour scar.

**Figure 1.**
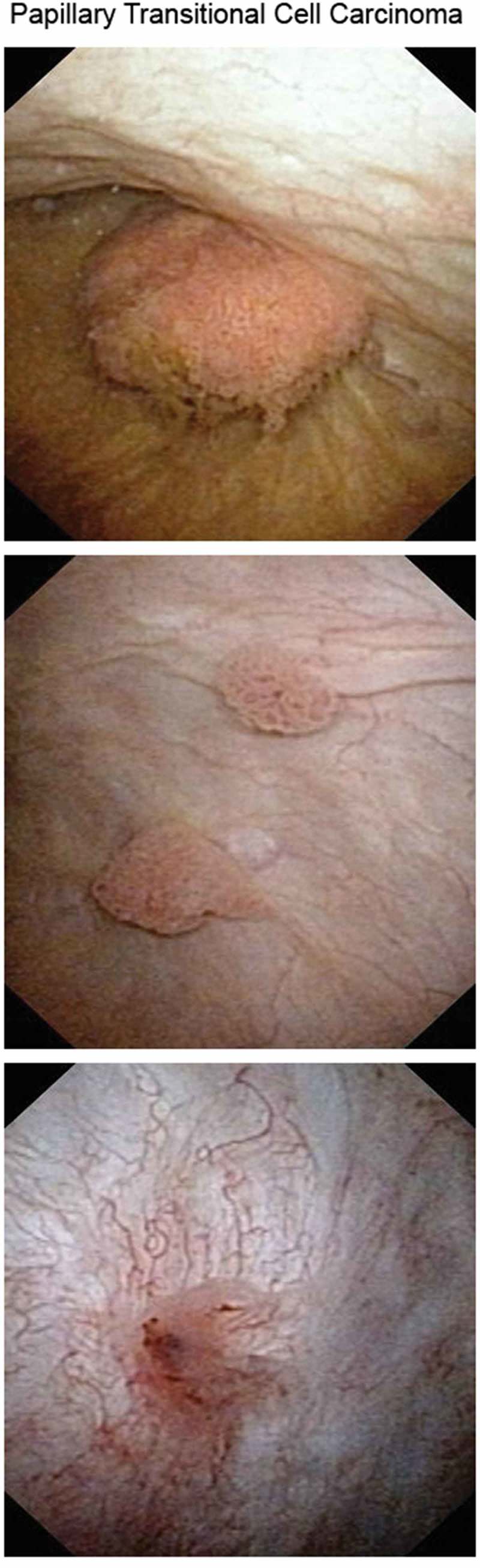
An example page from the PH.

Each consultant provided an image for every selected disorder, resulting in every pathological find being described with three different photographs. A short description accompanied the photographs. The PH was edited in PDF version and made available by download from the university server (Figure 1).

### Statistical analysis

Data were acquired and analysed by Statistics Open For All (SOFA) Statistics 1.4.6. A descriptive statistical analysis was performed to evaluate the sample characteristics and reported as mean ± standard deviation (SD) or as proportions. Statistical analysis of nominal variables comparing the two different JR groups using the chi-squared test was performed.

## Results

A total of 401 consecutive cystoscopies, of which 214 (53.4%) were performed by Group A and 187 (46.6%) by Group B, were considered. The mean (SD) age of patients was 72.1 (12) years, 96 (23.9%) were females and 305 (76.1%) males. In Group A, the median (SD) age was 73.0 (12.3) years and 171 (79.9%) of the patients were male, whilst in Group B the median (SD) age was 71.1 (11.6) years and 144 (77%) were males.

Group A was able to correctly identify the ureteric orifices, when possible, in 191/214 (89.3%) cases vs 131/187 (70.1%) in Group B (*P* < 0.01).

In Group A, cystoscopies were correctly marked as negative for suspicious tumoral masses in 152/168 patients (90.4%) and positive in 41/46 (89.1%), whilst in Group B 100/115 (87%) were correctly marked as negative for suspicious tumoral masses and 58/72 (80.6%) as positive (*P* = 0.35 and *P* = 0.22, respectively). Amongst positive cystoscopies, Group A found 54 lesions (81.8%) vs 66 found by the consultants, while Group B found 79 (78.2%) lesions vs 101 found by the consultants (*P* = 0.57).

**Figure 2.**
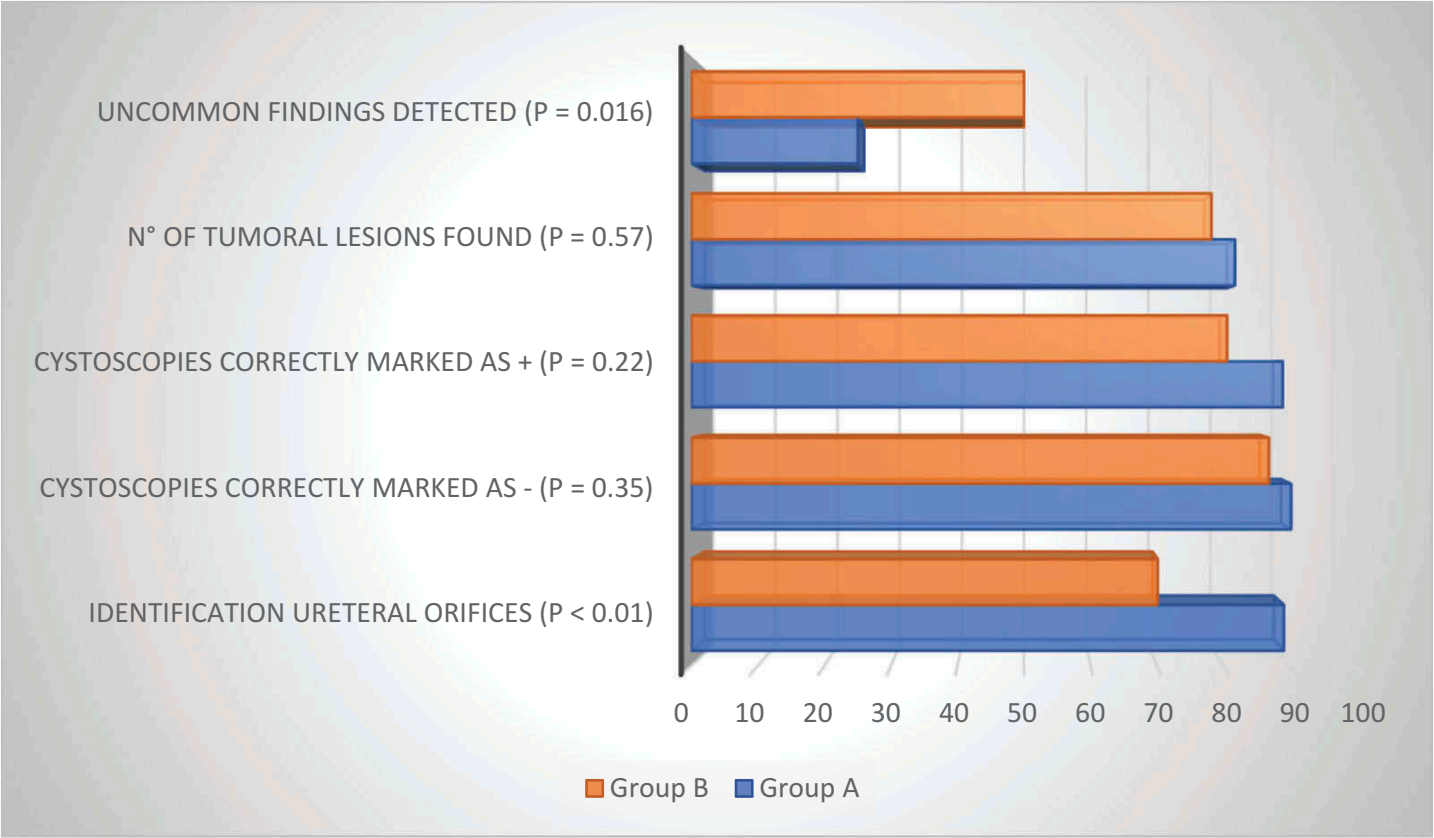
Comparative detections (%) between Group A and Group B. +, positive; -, negative.

Group B showed superior ability in the detection of uncommon findings (i.e., CIS, bullous oedema, interstitial cystitis, etc.) with 24/46 (52.2%) detected vs eight of 32 (25%) in Group A (*P* = 0.016). A case of interstitial cystitis was correctly identified by the JR who had studied the PH, but the JR who had not studied it failed to identify the pathology. Similarly, four of six cases of CIS were identified by Group B, whilst the only three cases in Group A were not recognised. The bullous oedema due to a permanent urethral catheter was correctly identified in eight of 10 cases by Group B and in two of five cases in Group A. All JRs rated the PH (Figure 1) as a very useful tool in theoretical and practical cystoscopy training (Figure 2).

## Discussion

In the last two decades the concept of teaching to reproduce what is done by and under the supervision of a tutor has been superseded. At the same time, the importance given to the learning curve needed in different diagnostic and therapeutic procedures to achieve adequate outcomes and results has increased. Many theoretical and practical training models have been introduced with the purpose of reducing the learning curve itself, in order to guarantee significant results in the shortest possible time [4–8]. In particular, in endourology, given the massive use of hands-on technology and instrumentation, we can find models of teaching and training aimed to improve the skills of residents and urologists in the use of these instruments and in the correct execution of procedures [9–13].

Diagnostic cystoscopy is the first and simplest endourological procedure performed by a JR, given the simplicity of execution and the rare possibility of harming the patient due to incorrect execution during the learning curve. Its use is fundamental not only for urological diagnostic purposes, but also for the education of medical students who can take advantage of the presence of a video interface to enhance learning of the anatomy of the urinary tract [14]. Its correct execution goes from an adequate knowledge of the anatomy of the lower urogenital tract to the functioning of the flexible cystoscope and manual ability of usage. An acceptable performance in terms of quality and correctness in execution, suitable duration of execution, and recognition of the pathology requires the performance of dozens of cases [15]. Some virtual computer-based simulators such as URO MENTOR™ (Simbionix Ltd, Lod, Israel) are specifically designed for self-training in performing the procedure. They allow users to become familiar with the use of the cystoscope and the steps to execute the procedure. They can also assess the level of experience achieved by the trainee [16,17].

In detail, Chou et al. [12] evaluated a virtual-reality (VR) simulator, which was found to be as effective as a bench simulator for teaching basic cystoscopy and ureteroscopy skills to novice trainers. The VR simulator can also reduce training costs and requires minimal teacher input. Mishra et al. [18] compared the use of a VR simulator and the use of a porcine model. They concluded that the overall usefulness of the models was similar. VR models were found to be more feasible for multiple tasking, but the lack of haptic feedback does not efficiently simulate the real conditions reported during endourological procedures. Hu et al. [3] conducted a study comparing the use of a model simulator and verbal instruction alone to achieve greater ureteroscopy and cystoscopy proficiency. The simulator training allowed students to achieve better results in their endourological skills.

It is interesting to note that although cystoscopy is mainly a urological diagnostic procedure, in the literature most of the simulation and teaching models proposed come from gynaecology, which may be due to a lack of confidence in execution, as a result of reduced exposure compared to that of urologists [19–23].

On the other hand, urology trainees have, from the start of their training in a urological department, daily access to dozens of cystoscopies, allowing them to observe and then perform under supervision. In a relatively short time, given the level of difficulty and chance of harming the patient through clumsy use of the instruments, constant access allows trainees the ability to achieve the practical skills they need to execute examinations correctly. For this reason, whilst taking into consideration that the various models of simulation proposed are undoubtedly useful [16,17,24,25], it is important to note however, that the literature is, above all, missing tools that can improve the sensitivity and specificity of the examination. In fact, ‘newbies’ might face problems with diagnostic/therapeutic indications in autonomy, especially when encountering unusual or unclear presentations.

PHs are tools recently introduced in the field of education in medicine. Their use in the education of residents and surgeons is a new concept. Currently, there are not any studies regarding PHs used as tools to train diagnostic skills in urology. Care et al. [26] developed a PH as a visual adjunct to detailed verbal discussion of surgical treatment options in patients with craniosynostosis. It consisted of pre- and postoperative photographs of patients. Their study showed that using selected photographs and presenting them in the context of discussion about therapeutics options could be helpful for families making decisions about surgery for their children.

The advantage of the PH, compared to other atlases, is that it is simple, immediate, comprised only of photographs, and easily consultable in few seconds from a mobile phone if there is any doubt during the execution of a procedure.

The proposed PH has proved to be a useful, low-cost and an easy to create tool, which is able to improve both the detection rate and the correct identification of some findings considered ‘rare’ during cystoscopy (bullous oedema, CIS, interstitial cystitis, etc.). In fact, in our present study, the JRs in Group B were able to correctly recognise 52.2% of the rare lesions vs only 25% of the JRs who had not studied the PH (*P* = 0.016). On the other hand, their manual inexperience in the use of the flexible cystoscope made them less able to detect the ureteric orifices and the exact number of bladder lesions.

The main bias of the study is that some pathological conditions, such as ureteric orifice duplication or ureterocoele, were not present in one or both groups. In the same way, the few JRs enrolled in the study (four vs four) does not exclude that the personal abilities and skills of a single JR might have influenced the results.

## Conclusions

Although JRs with previous experience had more confidence performing cystoscopy, which made them able to identify most of the ureteric orifices and suspicious lesions, they showed a significant inferiority in the detection of uncommon findings compared to the Group B JRs who had studied the PH. The PH was a useful tool for improving identification of pathological conditions, which could be used to enhance hands-on simulator and practical tutored training.
